# Supplementary material for: Serotonin modulates excitatory synapse maturation in the developing prefrontal cortex
Source: Nat Commun. 2024 Feb 16;15:1368. doi: 10.1038/s41467-024-45734-w (PMC10873381; doi:10.1038/s41467-024-45734-w)
Supplement: Supplementary file 1 — Supplementary Information [file 41467_2024_45734_MOESM1_ESM.pdf]

*Supplementary Information*

# Serotonin modulates excitatory synapse maturation in the developing prefrontal cortex

Roberto Ogelman[1], Luis E. Gomez Wulschner[1], Victoria M. Hoelscher[1], In-Wook Hwang[1], Victoria N. Chang[1], and Won Chan Oh[1,*]

[1]Department of Pharmacology, University of Colorado School of Medicine

Aurora, CO 80045, U.S.A.

*Corresponding author: Won Chan Oh

wonchan.oh@cuanschutz.edu

Classification: Article

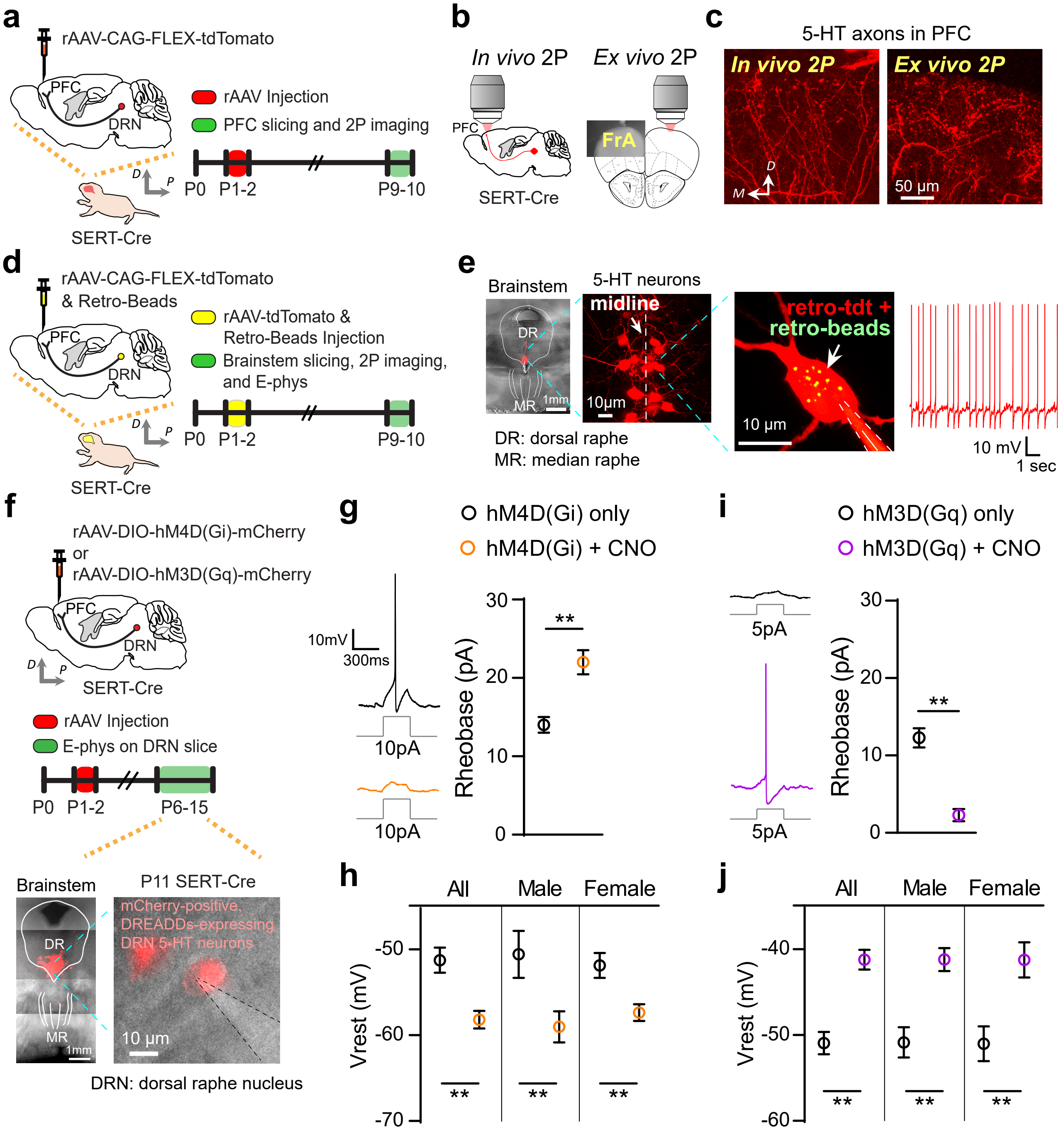


**Supplementary Fig. 1. Verification of CNO on rAAV-hM4D(Gi) or hM3D(Gq) expressing 5-HT neurons in acute brainstem slices from SERT-Cre mice. a, d, f**, Schematic of intracranial rAAV and/or retro-beads injections and experimental timeline. **b,** Diagram showing location of 2P *in vivo* and *ex vivo* imaging. **c,** Representative images of tdTomato expressing serotonergic axonal terminals in the PFC. **e,** DIC and 2P images of tdTomato and Retro-Beads expressing 5-HT neurons in the DRN. Spontaneous action potentials recorded in current-clamp mode. **g,** Example membrane potential traces evoked by current injection of 10 pA and quantative analysis of rheobase of mCherrry positive neurons after CNO bath application (30-60 min, 1 µM) in hM4D(Gi) expressing brainstem slices (Gi: n = 10 cells, 4 mice; Gi + CNO: n = 10 cells, 4 mice) **h,** Quantative analysis of resting membrane potential (Gi: n = 17 cells, 7 mice; Gi + CNO: n = 18 cells, 7 mice; 4 males, 3 females per group). **i,** Example traces evoked by 5 pA current injection and summary of rheobase of mCherrry positive neurons after CNO bath application (30-60 min, 1 µM) in hM3D(Gq) expressing brainstem slices (Gq: n = 11 cells, 4 mice; Gq + CNO: n = 11 cells, 4 mice). **j,** Quantative analysis of resting membrane potential (Gq: n = 16 cells, 6 mice; Gq + CNO: n = 15 cells, 6 mice; 3 males, 3 females per group). ***p* < 0.05; error bars represent SEM. Source data are provided as a Source Data file.

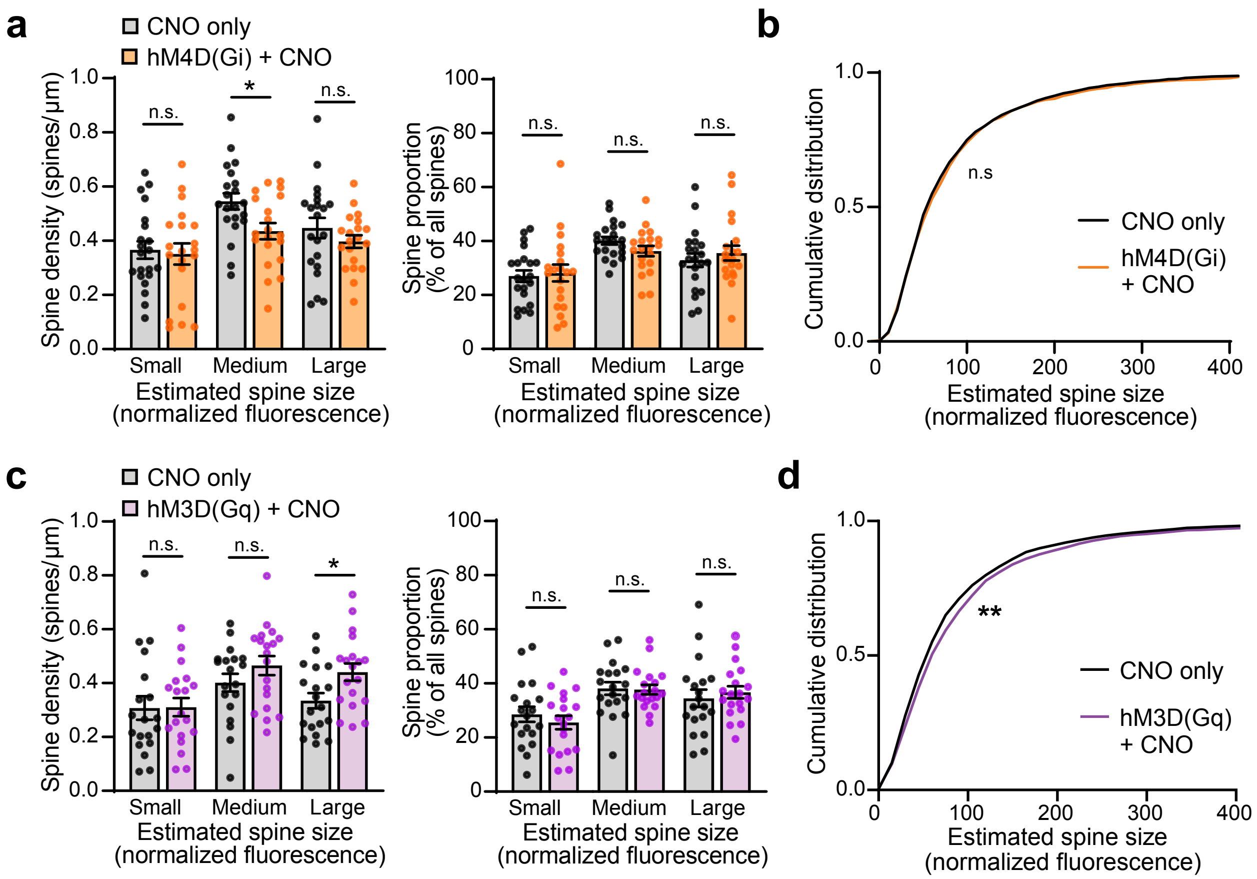


**Supplementary Fig. 2. Serotonin-mediated changes in spine density of PFC layer 2/3 pyramidal neurons *in vivo*. a,** Quantitative analysis of spine density and spine proportion by size (CNO only: n = 62 dendrites, 21 cells, 4 mice; hM4D(Gi) + CNO: 65 dendrites, 19 cells, 4 mice). **b,** Frequency distribution plot of all spine sizes (CNO only: n = 2255 spines, 21 cells, 4 mice; hM4D(Gi) + CNO: 1662 spines, 19 cells, 4 mice). **c,** Quantitative analysis of spine density and spine proportion by size (CNO only: n = 72 dendrites, 20 cells, 6 mice; hM3D(Gq) + CNO: 69 dendrites, 20 cells, 6 mice). **d,** Frequency distribution plot of all spine sizes (CNO only: n = 2327 spines, 20 cells, 6 mice; hM3D (Gq) + CNO: 2688 spines, 20 cells, 6 mice). $*p < 0.05$, $**p < 0.01$; error bars represent SEM. n.s., not significant. Source data are provided as a Source Data file.

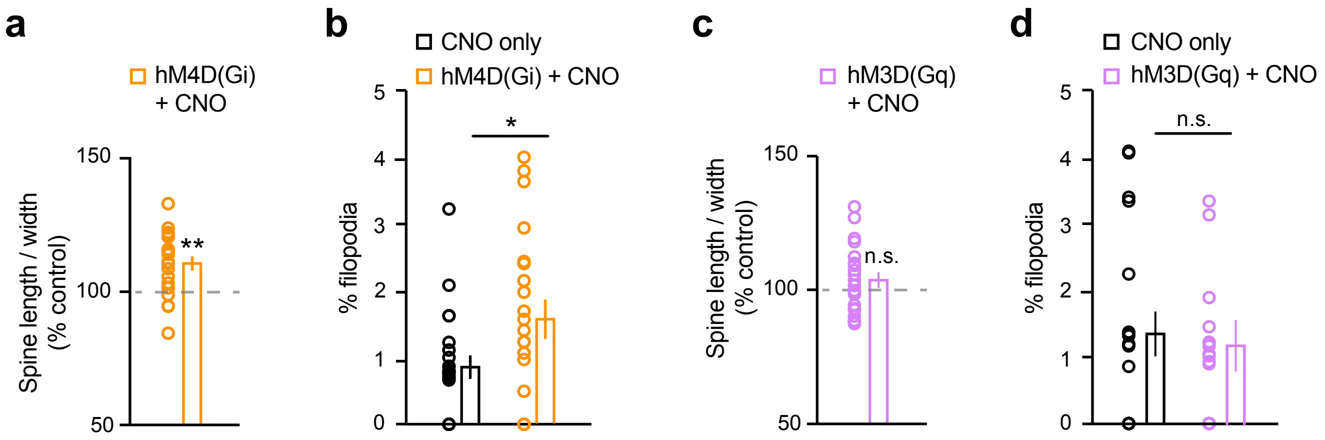


**Supplementary Fig. 3. Spine morphology analysis in DREADDs experiments.** Quantative analysis of **a,** spine length / width ratio and **b,** % filopodia in hM4D(Gi) experimental group (CNO only: n = 2762 spines, 21 cells; hM4D(Gi) + CNO: n = 1675 spines, 20 cells). Quantative analysis of **c,** spine length / width ratio and **d,** % filopodia in hM3D(Gq) experimental group (CNO only: n = 1333 spines, 18 cells; hM3D(Gq) + CNO: n = 1535 spines, 20 cells). *$p < 0.05$; error bars represent SEM. n.s., not significant. Source data are provided as a Source Data file.

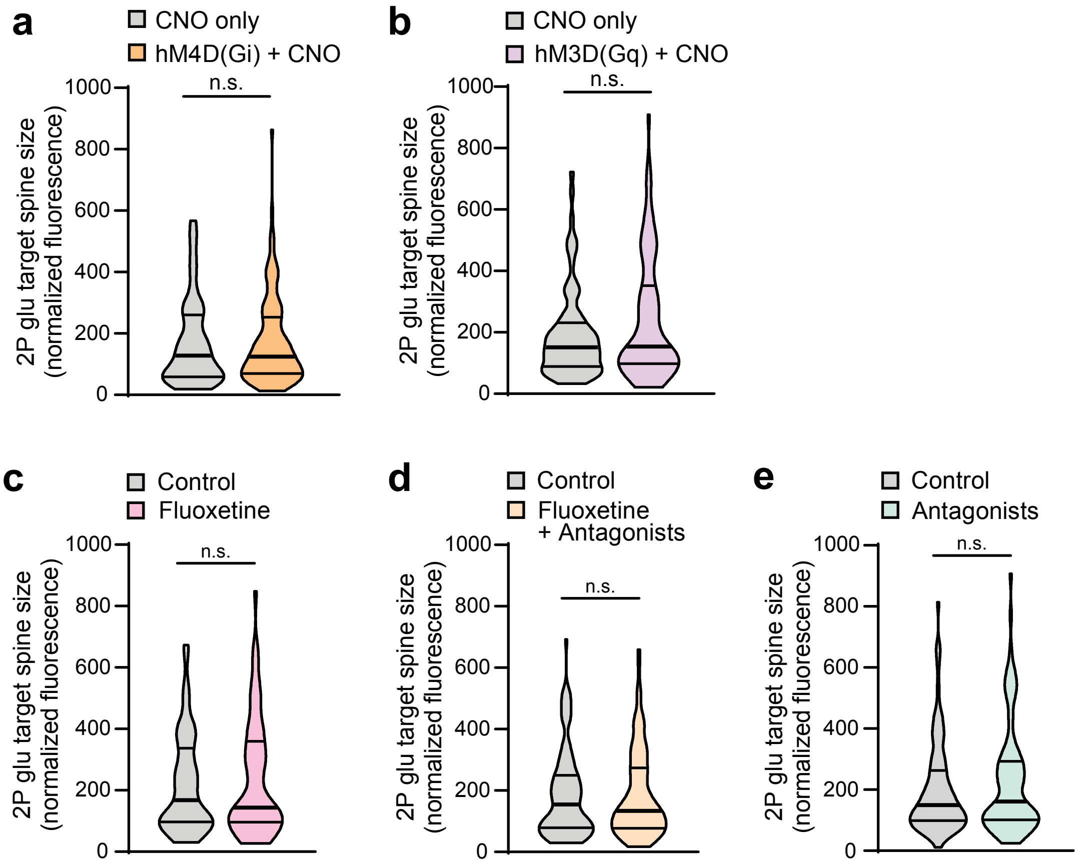


**Supplementary Fig. 4. Comparable target spine sizes for two-photon uEPSC measurements from *in vivo* manipulation experiments. a,** Violin plot distribution of spine sizes that uEPSCs were measured from in PFC slices of CNO only and hM4D(Gi) + CNO treated mice (CNO only: n = 80 spines, 12 cells, 4 mice; hM4D(Gi) + CNO: 115 spines, 12 cells, 4 mice). **b,** Violin plot distribution of spine sizes that uEPSCs were measured from in PFC slices of CNO only and hM3D(Gq) + CNO treated mice (CNO only: n = 79 spines, 13 cells, 6 mice; hM3D(Gq) + CNO: 83 spines, 14 cells, 6 mice). **c,** Violin plot distribution of spines that uEPSCs were measured from for control and FLX treated mice (Control: n = 59 spines, 9 cells, 3 mice; FLX: 64 spines, 11 cells, 3 mice). **d,** Violin plot distribution of spines that uEPSCs were measured from for control and FLX and antagonists treated mice (Control: n = 95 spines, 15 cells, 4 mice; FLX and Antagonists: 110 spines, 17 cells, 4 mice). **e,** Violin plot distribution of spines that uEPSCs were measured from for control and antagonists treated mice (Control: n = 93 spines, 15 cells, 4 mice; Antagonists: 93 spines, 12 cells, 4 mice). Violin plots: median ± upper and lower quartiles. n.s., not significant. Source data are provided as a Source Data file.

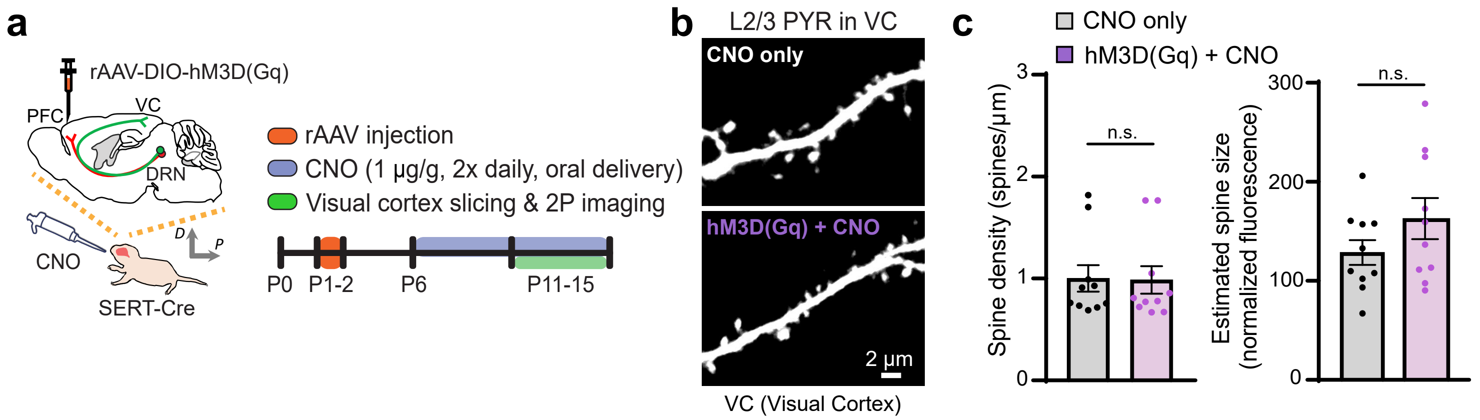


**Supplementary Fig. 5. rAAV-DREADD injection in the PFC does not affect excitatory synapse development on layer 2/3 pyramidal neurons in the developing visual cortex. a,** Schematic of rAAV injection in PFC, oral CNO delivery, and experimental timeline. **b,** Images from dendritic segments of layer 2/3 pyramidal neurons from visual cortex of CNO only and hM3D(Gq) and CNO treated mice. **c,** Quantitative analysis of spine density and size (CNO only: n = 37 dendrites, 10 cells, 3 mice; hM3D(Gq) + CNO: 37 dendrites, 10 cells. 3 mice). Error bars represent SEM. n.s., not significant. Source data are provided as a Source Data file.

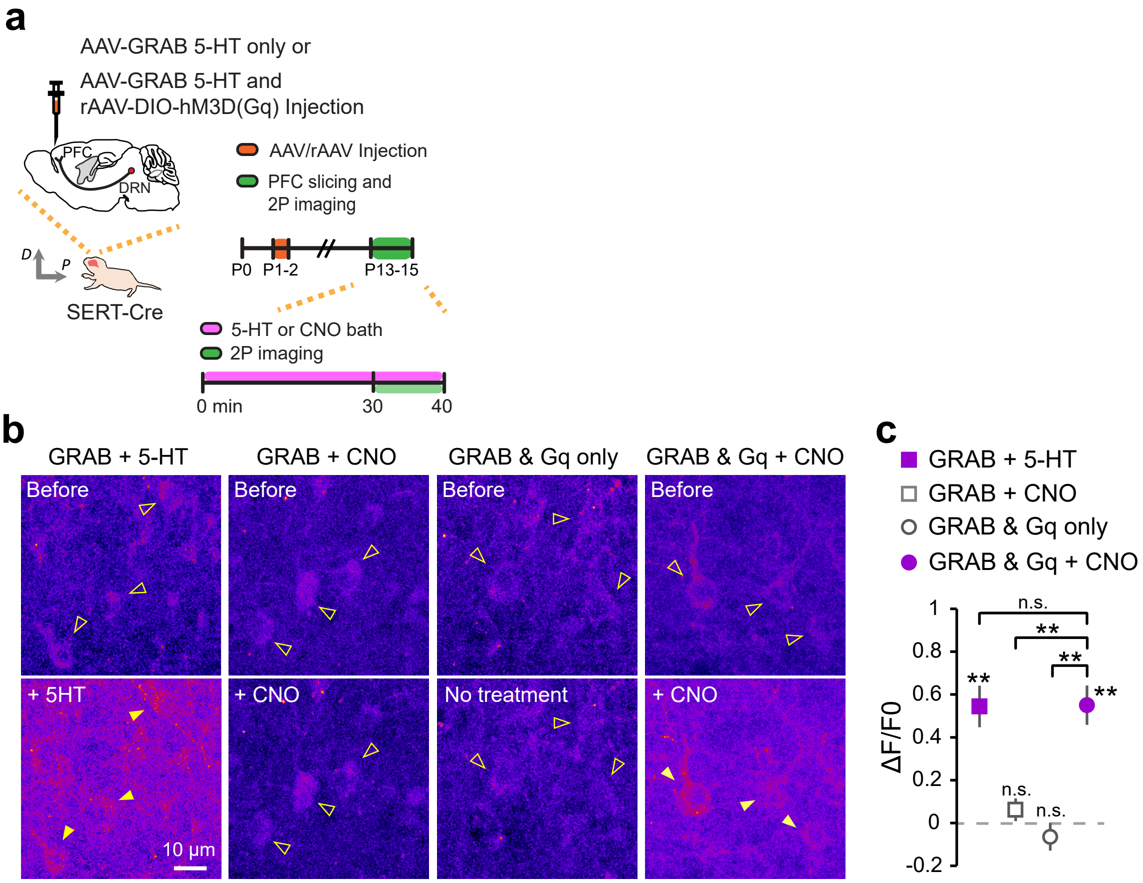


**Supplementary Fig. 6. Verificiation of 5-HT release in the PFC of rAAV-hM3D(Gq) injected mice using GRAB 5-HT1.0. a,** Schematic of intracranial AAV/rAAV injection and experimental timeline. **b,** Representative images of GRAB 5-HT expressing neurons from PFC layer 2/3 before and after 5-HT or CNO bath application (5-HT, 30-40 min, 10 µM; CNO, 30-40 min, 1 µM). **c**, Quantitative analysis of GRAB-5-HT fluorescent changes (GRAB + 5-HT: n = 42 cells, 2 mice; GRAB + CNO: n = 38 cells, 2 mice; GRAB & Gq, no treatment: n = 24 cells, 3 mice; GRAB & Gq + CNO: n = 94 cells, 3 mice). $^{**}p < 0.01$; error bars represent SEM. n.s., not significant. Source data are provided as a Source Data file.

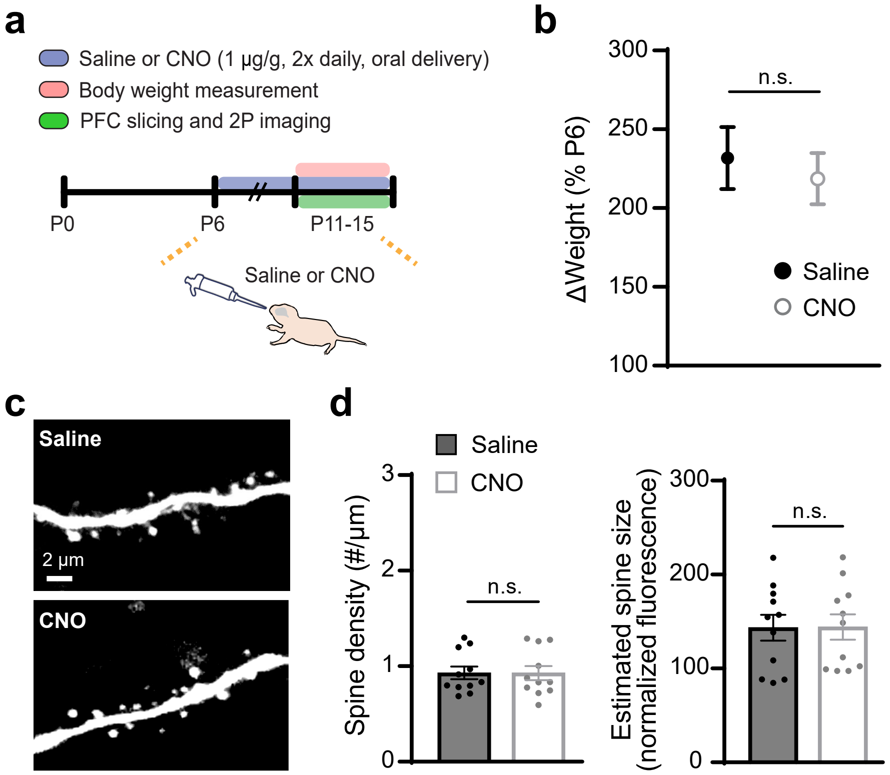


**Supplementary Fig. 7. *In vivo* CNO administration alone does not affect mouse health or dendritic spine development on layer 2/3 pyramidal neurons in the PFC. a,** Schematic of oral delivery of saline or CNO and timeline for pup weight measurements, acute PFC slicing, and 2-photon imaging, **b,** Quantative analysis of pup weight changes from start of saline or CNO delivery to time of experiments at P11-15 (saline: n = 4 mice; CNO: n = 4 mice). **c,** Representative 2-photon images of dendritic segments in saline and CNO conditions. **d,** Summary of spine density and size in saline and CNO treated mice (saline: n = 11 cells, 4 mice; CNO: n = 11 cells, 4 mice). Error bars represent SEM. n.s., not significant. Source data are provided as a Source Data file.

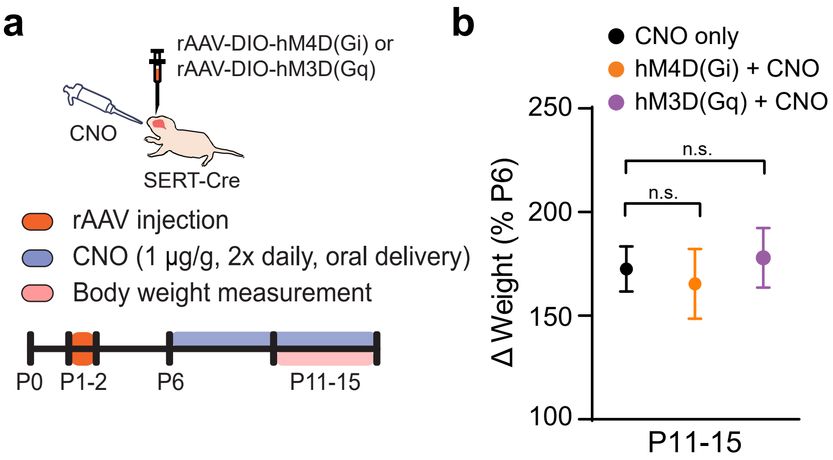


**Supplementary Fig. 8. rAAV-hM4D(Gi) or hM3D(Gq) injected mice show normal growth rates. a,** Schematic of rAAV injection, CNO oral delivery, and timeline for pup weight measurements. **b,** Summary of pup weight changes from start of CNO treatment to time of experiments at P11-15 (CNO only: n = 14 mice; hM4D(Gi) + CNO: 4 mice; hM3D(Gq) + CNO: 6 mice). Error bars represent SEM. n.s., not significant. Source data are provided as a Source Data file.

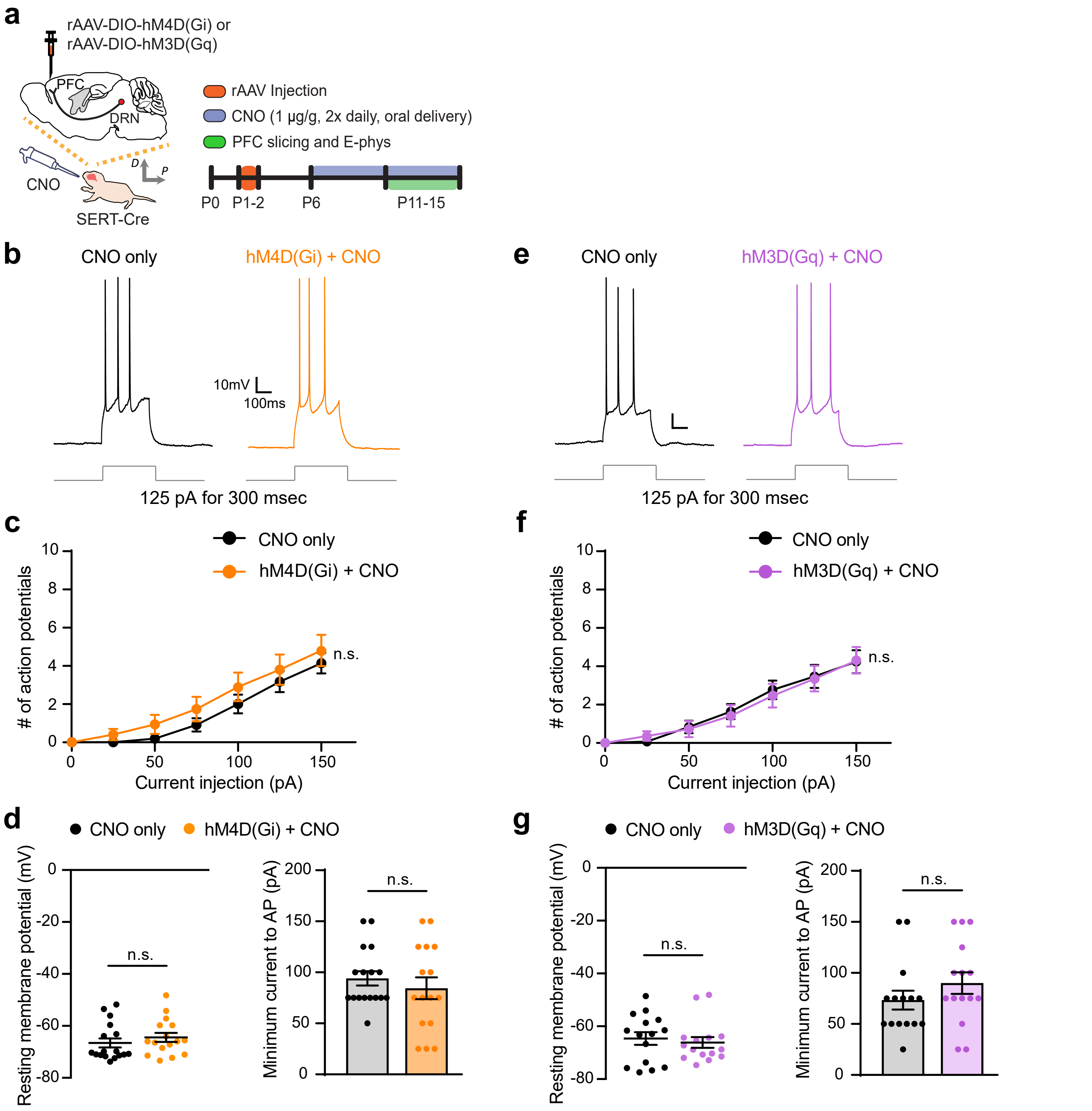


**Supplementary Fig. 9. Chemogenetic manipulation of PFC-projecting 5-HTergic neurons does not alter intrinsic properties of PFC layer 2/3 pyramidal neurons. a,** Schematic of rAAV injection, oral CNO delivery, and experimental timeline. **b,** Representative whole-cell traces of action potential firing evoked by current injection of 125 pA from CNO only and hM4D(Gi) and CNO treated mice. **c,** Quantative analysis of number of action potentials fired at increasing current injection steps. **d,** Quantative analysis of resting membrane potential and minimum current to elicit an action potential (CNO only: n = 17 cells, 3 mice; hM4D(Gi) + CNO: n = 16 cells, 3 mice). **e,** Representative traces of action potential firing evoked by current injection of 125 pA from CNO only and hM3D(Gq) and CNO treated mice. **f,** Quantative analysis of number of action potentials fired at increasing current injection steps. **g,** Summary of resting membrane potential and minimum current to elicit an action potential (CNO only: n = 15 cells, 3 mice; hM3D(Gq) + CNO: n = 15 cells, 3 mice). Error bars represent SEM. n.s., not significant. Source Data are provided as a Source Data file.

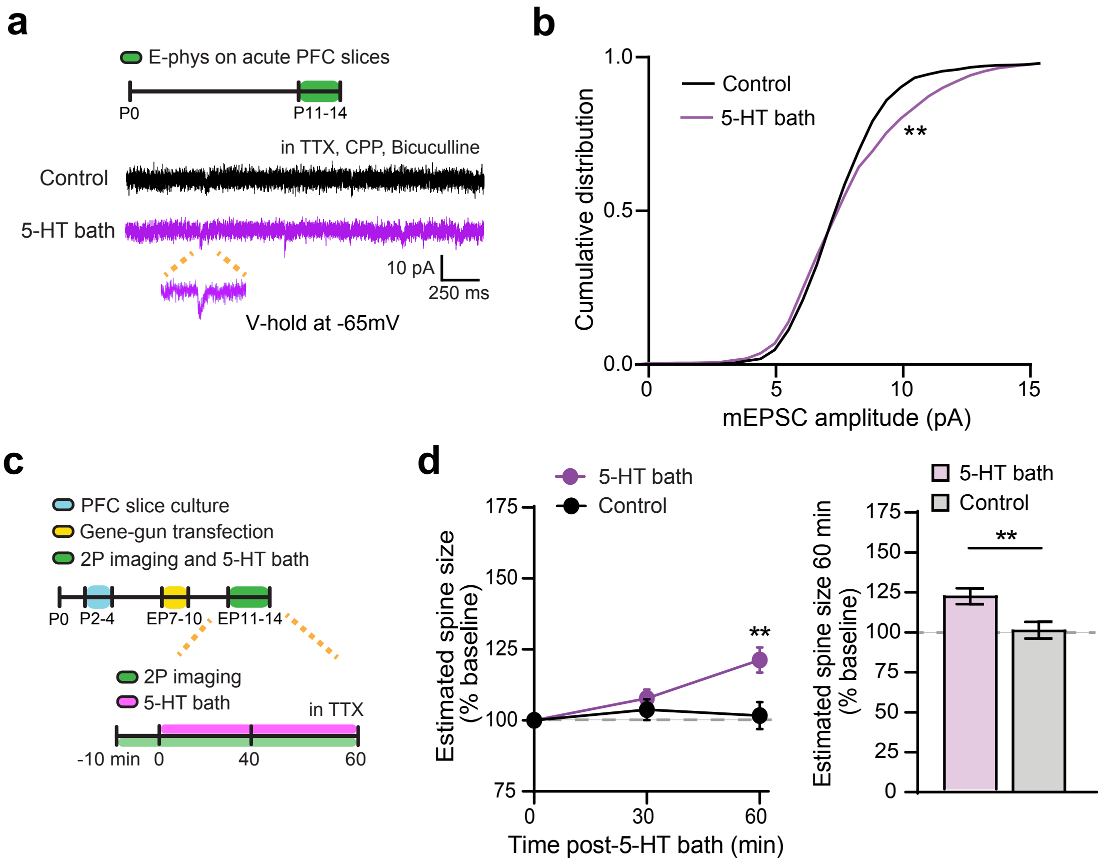


**Supplementary Fig. 10. Serotonin bath application induces cell-wide excitatory synaptic potentiation in the PFC. a,** Schematic of experimental timeline and representative mEPSC traces by whole-cell electrophysiological recordings. **b,** Frequency distribution plot of mEPSC amplitudes after 1 hr 5-HT bath application (Control: n = 11 cells; 5-HT: 9 cells, 10 µM). **c,** Schematic of experimental timeline. **d,** Time courses of spine size changes and quantitative analysis at 1 hr post 5-HT bath application (Control: n = 26 dendrites, 6 cells; 5-HT: 28 dendrites, 8 cells, 50 µM). $^{**}p < 0.01$; error bars represent SEM. Source data are provided as a Source Data file.

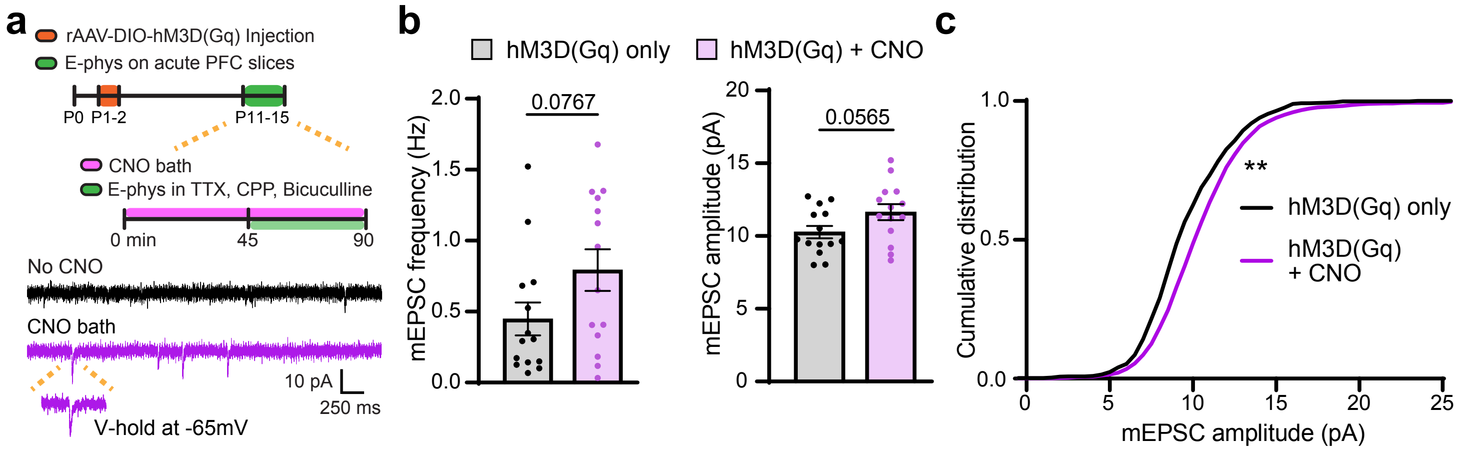


**Supplementary Fig. 11. hM3D(Gq)-mediated 5-HT release induces cell-wide excitatory synaptic potentiation in the PFC. a,** Schematic of rAAV injection, experimental timeline, and representative mEPSC traces. **b,** Quantative analysis of mEPSC frequency and amplitude recorded at -65 mV 45-90 min after CNO bath application (1 μM) (Gq only: n = 13 cells, 3 mice; Gq + CNO: 13 cells, 3 mice). **c,** Frequency distribution plot of mEPSC amplitudes. $^{**}p < 0.01$; error bars represent SEM. Source data are provided as a Source Data file.

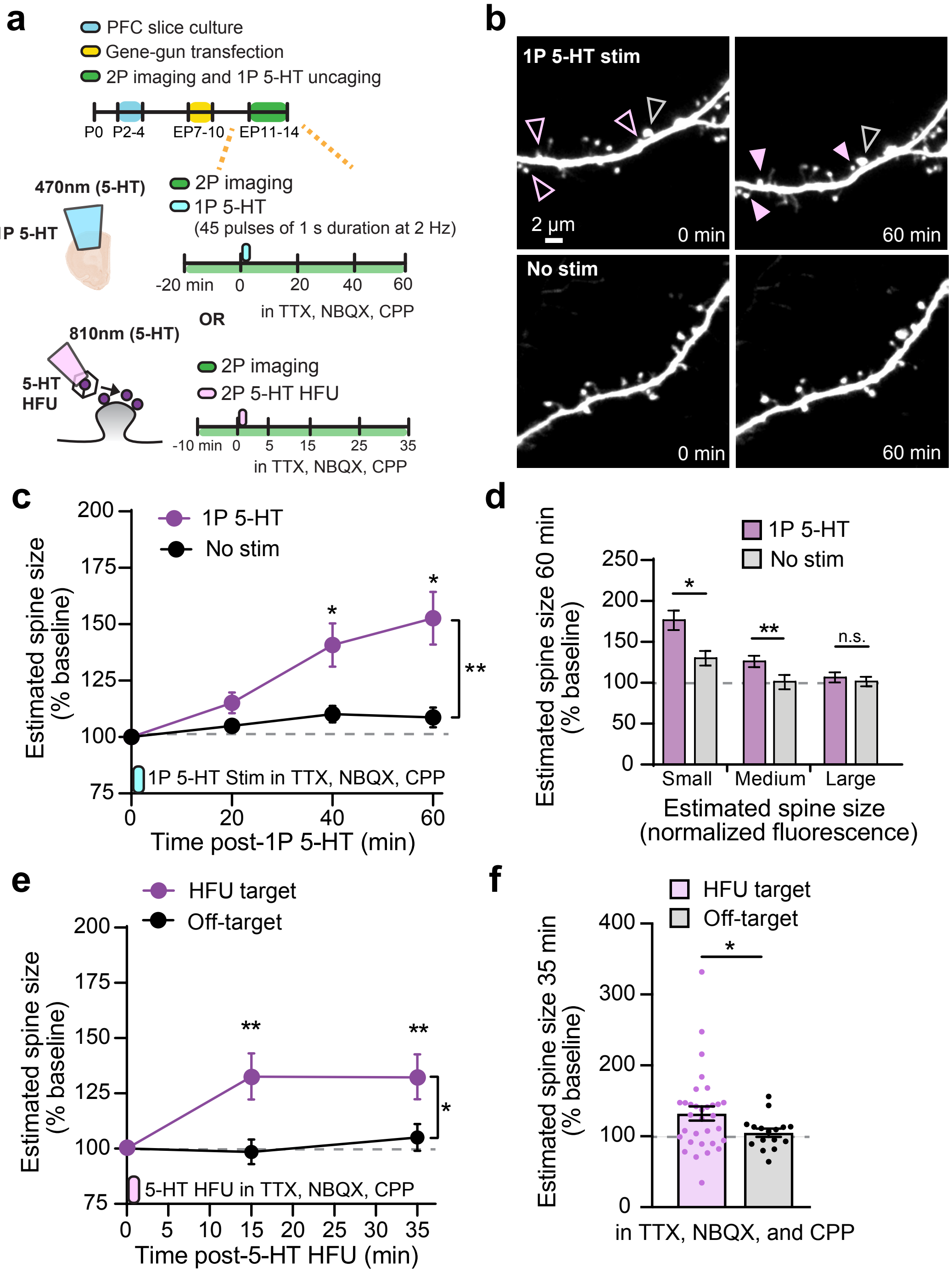


**Supplementary Fig. 12. 5-HT drives structural potentiation of dendritic spines independently of glutamatergic signaling. a,** Schematic of slice culture and experimental timeline for structural LTP after 1-photon (top) and 2-photon (bottom) 5-HT uncaging in TTX (1 µM), NBQX (10 µM), and CPP (10 µM). **b,** Two-photon images of dendrites with and without 1-photon 5-HT uncaging stimulation. Purple arrows indicate small and medium spines that underwent sLTP. Gray arrow shows a large spine that was unchanged. **c,** Time courses for spine size changes post 1-photon 5-HT uncaging. Blue indicator represents 1-photon 5-HT time point (Control: n = 20 dendrites, 5 cells; 1P 5-HT: 26 dendrites, 6 cells). **d,** Quantitative analysis of spine size change at 60 min, subdivided by spine size. **e,** Time courses for spine size changes following 5-HT HFU and **f,** summary of spine size at 35 min post 5-HT HFU. Purple indicator shows 2P 5-HT HFU time point (HFU target: n = 32 spines, 16 cells; Off-target: 16 ROIs, 16 cells). $*p < 0.05$, $**p < 0.01$; error bars represent SEM. n.s., not significant. Source data are provided as a Source Data file.

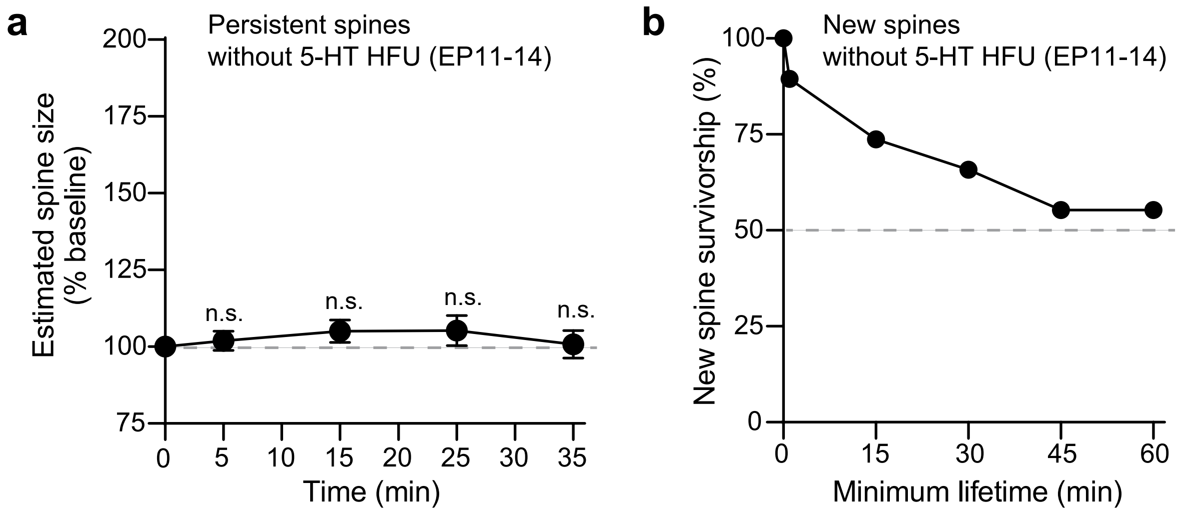


**Supplementary Fig. 13. Dynamics of dendritic spines of PFC layer 2/3 pyramidal neurons in RuBi-5-HT and TTX containing ACSF. a,** Time course for spine size changes of no stim control spines (n = 20 dendrites, 10 cells). **b,** Time course for new spine survivorship of no stim control spines (n = 38 spines, 7 cells). Error bars represent SEM. n.s., not significant. Source data are provided as a Source Data file.

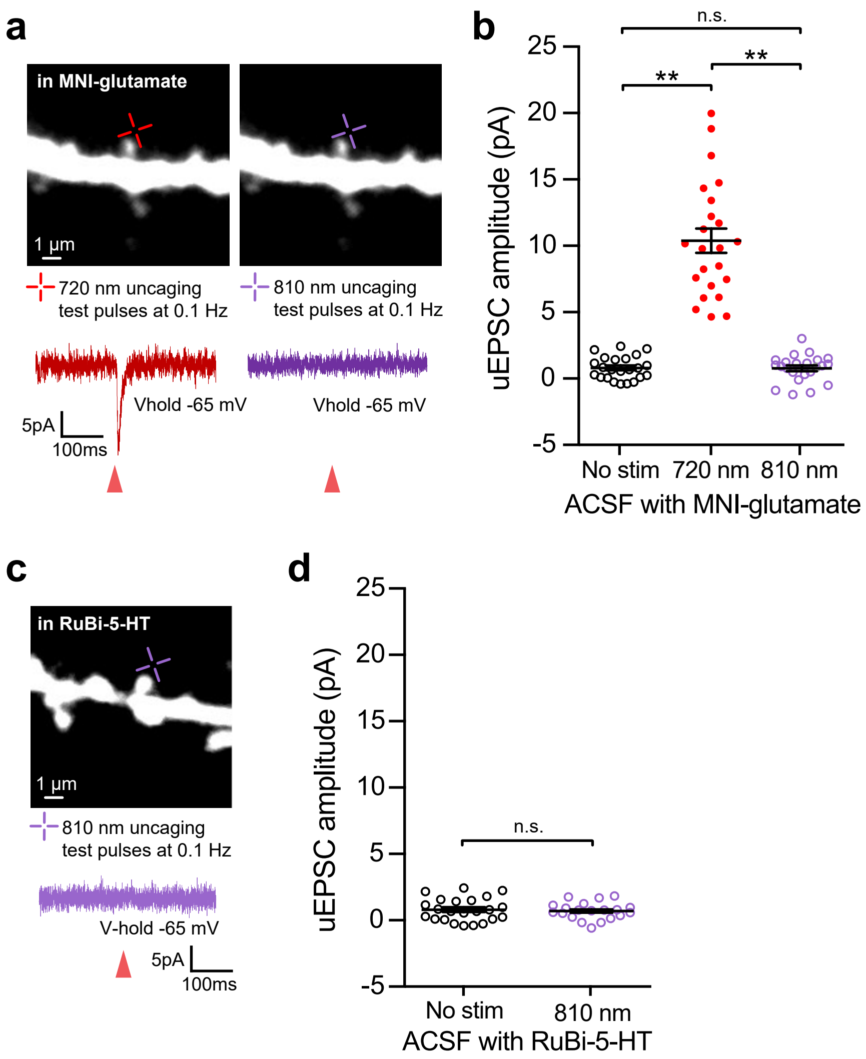


**Supplementary Fig. 14. Two-photon laser wavelength specific uncaging.**

**a,** Representative 2-photon images and uEPSC traces from the same spine evoked by 720 nm (left) or 810 nm (right) in MNI-glutamate (2.5 mM) containing ACSF (red cross represents 720 nm uncaging point, purple cross represents 810 nm uncaging point; red arrows indicate uncaging time point). **b,** Summary of uEPSCs (No stim: n = 23 off-stim responses, 5 cells; 720 nm: n = 23 spines, 5 cells; 810 nm: n = 23 spines, 5 cells). **c,** A representative image and an uncaging response recorded at -65 mV by 810 nm laser in RuBi-5-HT (0.1 mM) containing ACSF (purple cross represents 810 nm uncaging point; red arrows indicate uncaging time point). **d,** Summary data (No stim: n = 23 off-stim responses, 5 cells; 810 nm: n = 20 spines, 2 cells). $^{**}p < 0.01$; error bars represent SEM. n.s., not significant. Source data are provided as a Source Data file.

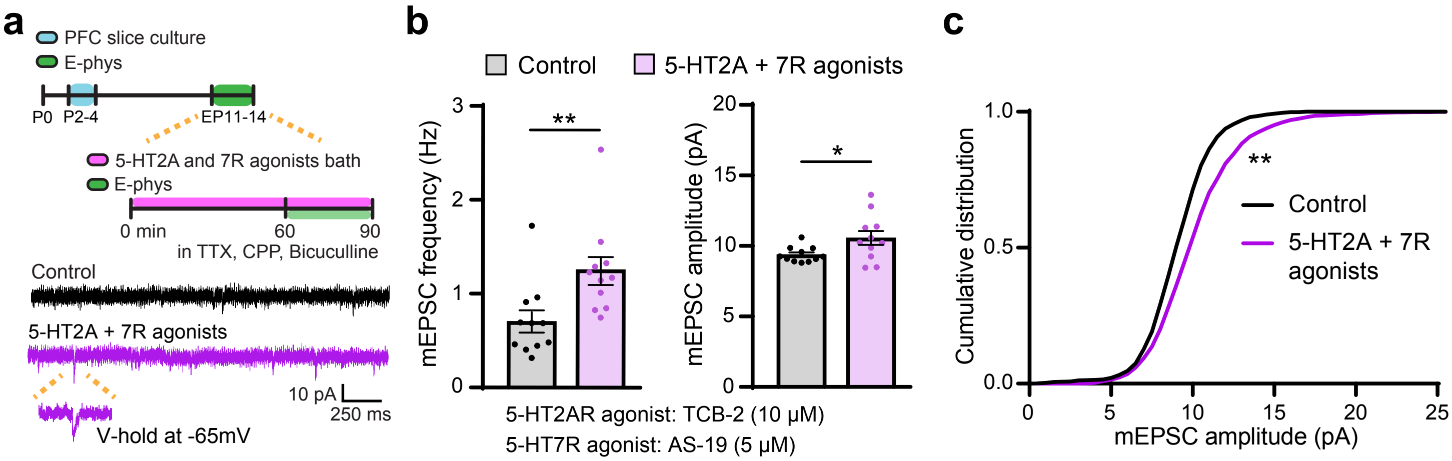


**Supplementary Fig. 15. 5-HT2A and 5-HT7 receptor agonist bath application increases mEPSC frequency and amplitude on PFC layer 2/3 pyramidal neurons. a,** Schematic of experimental timeline and representative mEPSC traces by whole-cell electrophysiological recordings after bath application of 5-HT2AR agonist (TCB-2, 10 μM) and 5-HT7R agonist (AS-19, 5 μM). **b,** Quantative analysis of mEPSC frequency and amplitude recorded 60-90 min after bath application of agonists (Control: n = 11 cells; TCB-2 + AS-19: n = 11 cells). **c,** Frequency distribution plot of mEPSC amplitudes. $*p < 0.05$, $**p < 0.01$; error bars represent SEM. Source data are provided as a Source Data file.

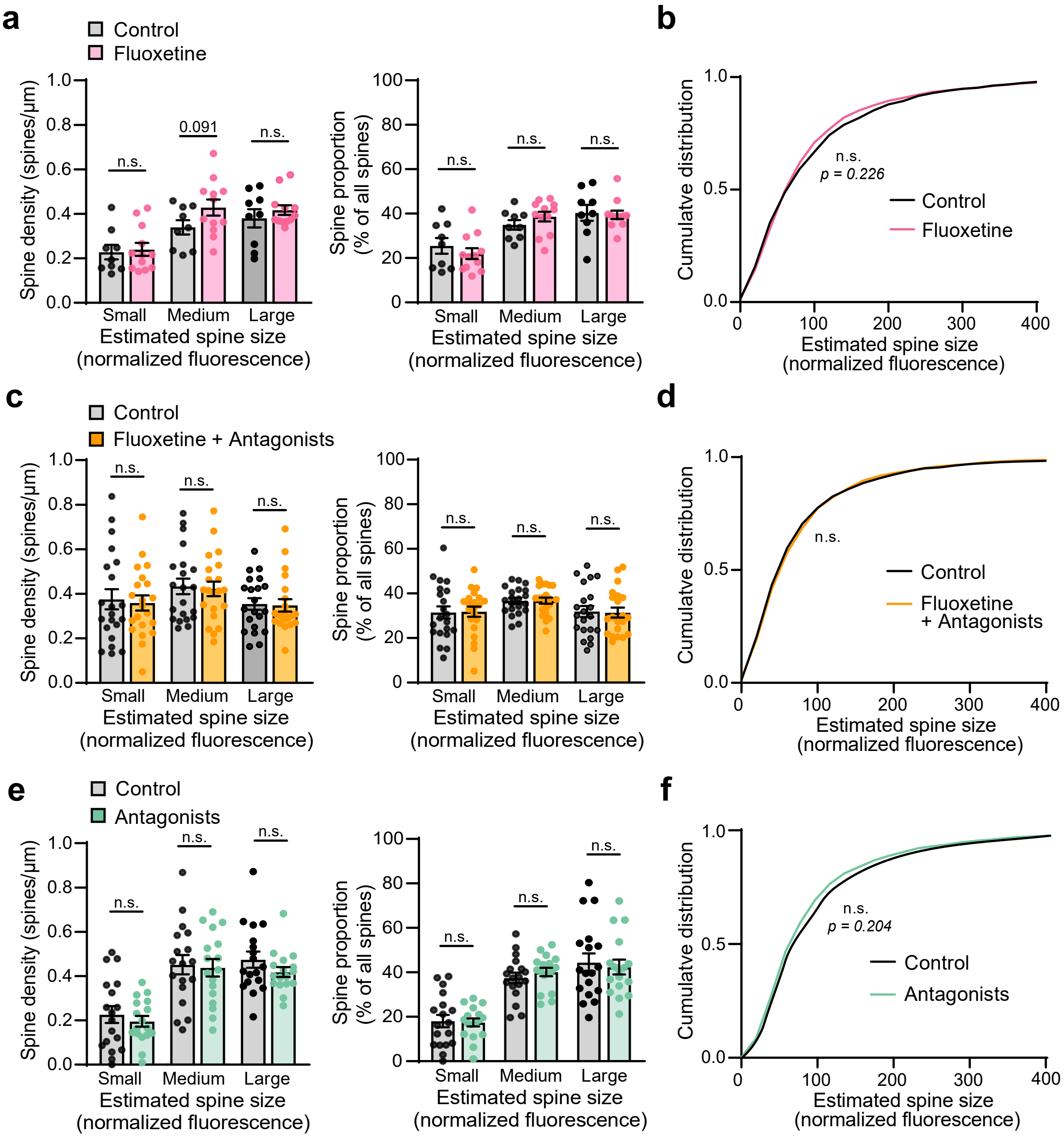


**Supplementary Fig. 16. Spine density changes by *in vivo* treatment of fluoxetine, fluoxetine and 5-HT2AR and 5-HT7R antagonists, or 5-HT2AR and 5-HT7R antagonists only. a,** Quantitative analysis of spine density and spine proportion by size after oral FLX exposure (Control: n = 50 dendrites, 13 cells, 5 mice; FLX: 69 dendrites, 18 cells, 5 mice). **b,** Frequency distribution plot of all spine sizes (Control: n = 792 spines, 13 cells, 5 mice; FLX: 1417 spines, 18 cells, 5 mice). **c**, Summary of spine density and spine proportion by size after FLX and 5-HT2AR and 5-HT7R antagonists treatment (Control: n = 64 dendrites, 21 cells, 4 mice; FLX + Antagonists: 67 dendrites, 21 cells, 4 mice). **d,** Frequency distribution plot of all spine sizes (Control: n = 2131 spines, 21 cells, 4 mice; FLX + Antagonists: 2180 spines, 21 cells, 4 mice). **e,** Quantitative analysis of spine density and spine proportion by size after oral 5-HT2AR and 5-HT7R antagonists treatment (Control: n = 60 dendrites, 18 cells, 4 mice; Antagonists: 60 dendrites, 17 cells, 4 mice). **f,** Frequency distribution plot of all spine sizes (Control: n = 2188 spines, 18 cells, 4 mice; Antagonists: 1797 spines, 17 cells, 4 mice). Error bars represent SEM. n.s., not significant. Source data are provided as a Source Data file.

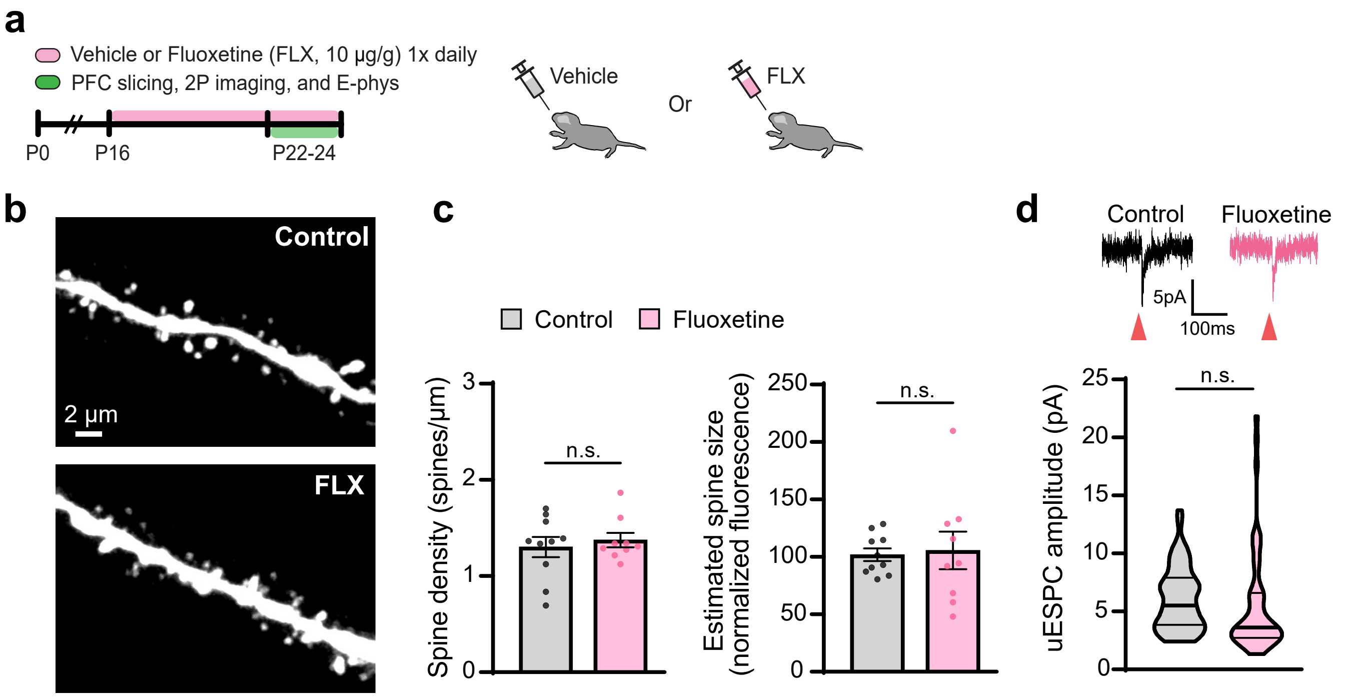


**Supplementary Fig. 17. Fluoxetine administration to old pups aged P16-24 does not alter excitatory synapses on layer 2/3 pyramidal neurons in the PFC. a,** Schematic of experimental timeline and oral FLX adminstration. **b,** 2-photon images of dendrites from PFC layer 2/3 pyramidal neurons from vehicle control and FLX treated mice. Quantitative analysis of **c**, spine density and size (Control: n = 31 dendrites, 10 cells, 3 mice; FLX: 27 dendrites, 9 cells, 3 mice) and **d,** uEPSCs recorded at -65 mV (Control: n = 59 spines, 10 cells, 3 mice; FLX: 50 spines, 9 cells, 3 mice; red arrows indicate 2P uncaging time point). Violin plots: median ± upper and lower quartiles. Error bars represent SEM. n.s., not significant. Source data are provided as a Source Data file.

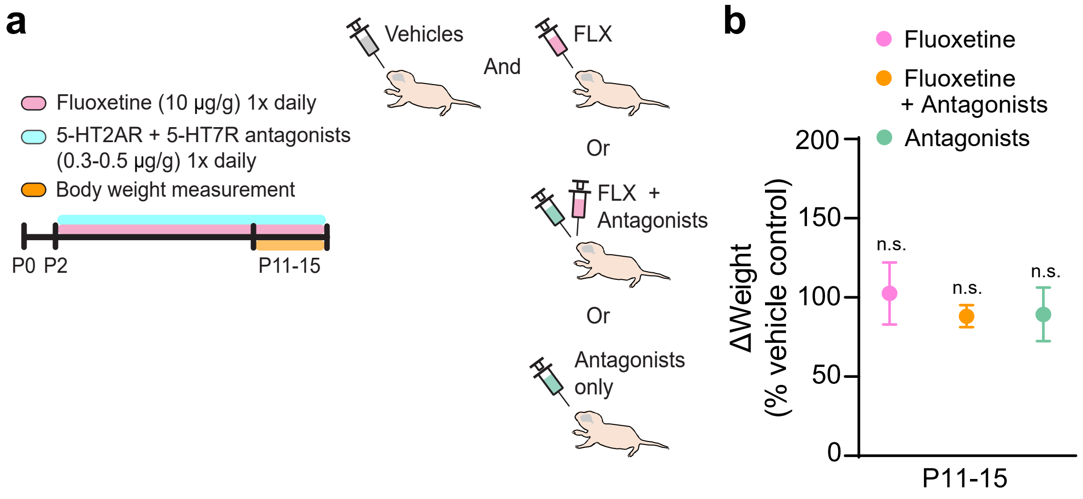


**Supplementary Fig. 18. Fluoxetine only, fluoxetine and 5-HT2AR and 5-HT7R antagonists, or 5-HT2AR and 5-HT7R antagonists only exposed pups show normal growth rates. a,** Schematic of FLX, FLX and antagonists, or antagonists only oral delivery and timeline for pup weight measurements. **b,** Pup weight changes compared to age, litter, and gender-matched control pups from start of FLX, FLX and antagonists, or antagonists only exposure to time of experiments (Control: n = 9 mice, FLX: 5 mice, FLX + Antagonists: 4 mice, Antagonists only: 4 mice). Error bars represent SEM. n.s., not significant. Source data are provided as a Source Data file.

| Treatment | Total # of spines | # of spines that underwent enlargement (sLTP) | % of spines that underwent enlargement (sLTP) | Spine size changes (% baseline) |
|---|---|---|---|---|
| **No stim (EP11-14)** | 337 | 137/337 | 40.7 % | 100.76 % ± 4.50 |
| **HFU target (EP11-14)** | 30 | 21/30 | 70.0 % | 140.13 % ± 11.42 |
| **Off-target (EP11-14)** | 151 | 61/151 | 40.7 % | 103.86 % ± 3.60 |
| **HFU target (EP15-20)** | 22 | 11/22 | 50.0 % | 112.65 % ± 6.21 |
| **5-HT HFU (shift)** | 22 | 9/22 | 40.9 % | 102.04 % ± 15.85 |
| **LFU target (0.1Hz)** | 23 | 12/23 | 52.2 % | 110.38 % ± 5.43 |
| **5-HT HFU (Layer V)** | 28 | 14/28 | 50.0 % | 113.78 % ± 11.43 |
| **HFU target + MDL** | 18 | 7/18 | 38.9 % | 96.95 % ± 7.67 |
| **Off-target in MDL** | 52 | 19/52 | 36.5 % | 100.76 % ± 3.35 |
| **HFU target + Gö** | 15 | 8/15 | 53.3 % | 119.11 % ± 16.38 |
| **Off-target in Gö** | 187 | 81/187 | 43.3 % | 104.04 % ± 5.11 |
| **HFU target + SB** | 18 | 9/18 | 50.0 % | 117.78 % ± 12.19 |
| **Off-target in SB** | 102 | 45/102 | 44.1 % | 104.54 % ± 7.25 |
| **HFU target 0mM Ca** | 20 | 10/20 | 50.0 % | 99.92 % ± 7.22 |
| **Off-target 0mM Ca** | 98 | 43/98 | 43.8 % | 101.75 % ± 4.99 |
| **HFU target in MDL + DR** | 20 | 9/20 | 45.0 % | 114.10 % ± 10.41 |
| **Off-target in MDL + DR** | 94 | 34/94 | 36.2 % | 106.94 % ± 6.75 |
| **HFU target in NBQX/CPP** | 32 | 22/32 | 68.8 % | 132.21 % ± 9.98 |
| **Off-target in NBQX/CPP** | 140 | 77/140 | 55.0 % | 105.08 % ± 5.75 |

**Supplementary Table 1. Two-photon 5-HT HFU increases the proportion of spines that undergo sLTP.** Summary of the number and percentage of spines that underwent sLTP at 35 min post-stim for 2-photon 5-HT HFU sLTP data. Spine size changes in each of experimental conditions are also shown for reference. Source data are provided as a Source Data file.

| Treatment | Estimated initial spine size (+/- SEM) |
|---|---|
| **HFU target (EP11-14)** | 124.55 ± 12.54 |
| **5-HT HFU (shift)** | 127.75 ± 21.99 |
| **HFU target (EP15-20)** | 89.02 ± 11.82 |
| **LFU target (0.1Hz)** | 115.53 ± 14.23 |
| **5-HT HFU (Layer V)** | 84.78 ± 10.38 |
| **HFU target + MDL** | 121.78 ± 7.79 |
| **HFU target + Gö** | 86.92 ± 10.56 |
| **HFU target + SB** | 115.57 ± 13.95 |
| **HFU target 0mM Ca** | 123.72 ± 20.31 |
| **HFU target in MDL + DR** | 89.14 ± 10.91 |
| **HFU target in NBQX/CPP** | 115.34 ± 13.65 |

n.s.

**Supplementary Table 2**. **Initial spine sizes are comparable for all two-photon 5-HT uncaging sLTP target spines.** Summary of initial spine sizes for all stimulated target spines for all 5-HT HFU sLTP experimental conditions. One-way ANOVA; n.s., not significant. Source data are provided as a Source Data file.

| Groups | Initial SEPGluA2 Expression (+/- SEM) | |
|---|---|---|
| **Persistent Spines (no drug)** | 0.588 ± 0.059 | n.s. (vs. MDL + SB); ** (vs. Newly formed spines) |
| **Persistent Spines (MDL + SB)** | 0.527 ± 0.061 | |
| **Newly formed spines** | 0.335 ± 0.043 | |

**Supplementary Table 3**. **Initial SEP-GluA2 expression of 5-HT HFU target spines.** Summary of initial SEP-GluA2 for all stimulated target spines for 5-HT HFU experimental conditions. $^{**}p < 0.01$; n.s., not significant. Source data are provided as a Source Data file.
