# Supplementary material for: Serotonin modulates excitatory synapse maturation in the developing prefrontal cortex
Source: Nat Commun. 2024 Feb 16;15:1368. doi: 10.1038/s41467-024-45734-w (PMC10873381; doi:10.1038/s41467-024-45734-w)
Supplement: Supplementary file 3 — Inventory of Supporting Information [file 41467_2024_45734_MOESM3_ESM.pdf]

Serotonin modulates excitatory synapse maturation in the developing prefrontal cortex

Roberto Ogelman[1], Luis E. Gomez Wulschner[1], Victoria M. Hoelscher[1], In-Wook Hwang[1], Victoria N. Chang[1], and Won Chan Oh[1,*]

[1]Department of Pharmacology, University of Colorado School of Medicine
Aurora, CO 80045, U.S.A.

Inventory of Supporting Information

1. Supplementary Figs. 1-18 (and Figure Legends)

2. Supplementary Tables 1-3 (and Table Captions)

3. Supplementary Video 1
