# Supplementary material for: Serotonin modulates excitatory synapse maturation in the developing prefrontal cortex
Source: Nat Commun. 2024 Feb 16;15:1368. doi: 10.1038/s41467-024-45734-w (PMC10873381; doi:10.1038/s41467-024-45734-w)
Supplement: Supplementary file 5 — Reporting Summary [file 41467_2024_45734_MOESM5_ESM.pdf]

Corresponding author(s): Won Chan Oh
Last updated by author(s): Jan 15, 2024

Reporting Summary

Nature Portfolio wishes to improve the reproducibility of the work that we publish. This form provides structure for consistency and transparency in reporting. For further information on Nature Portfolio policies, see our Editorial Policies and the Editorial Policy Checklist.

Statistics

For all statistical analyses, confirm that the following items are present in the figure legend, table legend, main text, or Methods section.

n/a	Confirmed	
☐	☒	The exact sample size (n) for each experimental group/condition, given as a discrete number and unit of measurement
☒	☐	A statement on whether measurements were taken from distinct samples or whether the same sample was measured repeatedly
☐	☒	The statistical test(s) used AND whether they are one- or two-sided<br>*Only common tests should be described solely by name; describe more complex techniques in the Methods section.*
☒	☐	A description of all covariates tested
☐	☒	A description of any assumptions or corrections, such as tests of normality and adjustment for multiple comparisons
☐	☒	A full description of the statistical parameters including central tendency (e.g. means) or other basic estimates (e.g. regression coefficient) AND variation (e.g. standard deviation) or associated estimates of uncertainty (e.g. confidence intervals)
☐	☒	For null hypothesis testing, the test statistic (e.g. F, t, r) with confidence intervals, effect sizes, degrees of freedom and P value noted<br>*Give P values as exact values whenever suitable.*
☒	☐	For Bayesian analysis, information on the choice of priors and Markov chain Monte Carlo settings
☒	☐	For hierarchical and complex designs, identification of the appropriate level for tests and full reporting of outcomes
☒	☐	Estimates of effect sizes (e.g. Cohen's d, Pearson's r), indicating how they were calculated

Our web collection on statistics for biologists contains articles on many of the points above.

Software and code

Policy information about availability of computer code

Data collection	No custom-written software was used in data collection. All image and electrophysiology data were collected by open source [Prairie View (Bruker), MultiClamp 700B (Molecular Devices)].
Data analysis	All data was analyzed by open-source software [ImageJ (NIH), Clampfit 10.3 (Molecular Devices), and OriginPro 8.5 (OriginLab)] as stated in the manuscript.

For manuscripts utilizing custom algorithms or software that are central to the research but not yet described in published literature, software must be made available to editors and reviewers. We strongly encourage code deposition in a community repository (e.g. GitHub). See the Nature Portfolio guidelines for submitting code & software for further information.

Data

Policy information about availability of data

All manuscripts must include a data availability statement. This statement should provide the following information, where applicable:

- Accession codes, unique identifiers, or web links for publicly available datasets
- A description of any restrictions on data availability
- For clinical datasets or third party data, please ensure that the statement adheres to our policy

We declare that all data supporting the findings of this study are provided within the paper and its supplementary information. Source data generated in this study are provided in the Source Data file.

Research involving human participants, their data, or biological material

Policy information about studies with human participants or human data. See also policy information about sex, gender (identity/presentation), and sexual orientation and race, ethnicity and racism.

Reporting on sex and gender	N/A, No human date were used.
Reporting on race, ethnicity, or other socially relevant groupings	N/A, No human date were used.
Population characteristics	N/A, No human date were used.
Recruitment	N/A, No human date were used.
Ethics oversight	N/A, No human date were used.

Note that full information on the approval of the study protocol must also be provided in the manuscript.

Field-specific reporting

Please select the one below that is the best fit for your research. If you are not sure, read the appropriate sections before making your selection.

☒ Life sciences ☐ Behavioural & social sciences ☐ Ecological, evolutionary & environmental sciences

For a reference copy of the document with all sections, see nature.com/documents/nr-reporting-summary-flat.pdf

Life sciences study design

All studies must disclose on these points even when the disclosure is negative.

Sample size	Data from a minimum of 3 independent slice culture preparations (average of 6) or a minimum of 3 mice per each group for acute slice work were used for each experimental comparison. All statistics were calculated across cells. Error bars represent standard error of the mean and significance was set at P = 0.05 (Student's two-tailed t test, non-parametric Mann-Whitney, or one way ANOVA). Survival rate across conditions for individual time points was compared by Fisher's exact test (two-sided) or using a log-rank test for survival comparisons throughout the entire imaging period. Outliers were not excluded from the data. No statistical methods were used to pre-determine sample sizes but the sample sizes used in our study, such as the number of neurons and animals, are about the same or more compared to the previous publication (Oh et al., PNAS, 2013; Oh et al., Science, 2016; Kleinjan et al., Neuron, 2023).
Data exclusions	Outliers were not excluded from the data. All data were included for analysis and figures. No data were excluded.
Replication	Replication was successful by at least 3 authors blindly.
Randomization	All mice were randomly allocated to the different experimental conditions. Individual dendritic spines were randomly targeted for experiments (spines were 50-100 µm from the soma on secondary and/or tertiary apical dendrites).
Blinding	Blind data analysis was conducted in in vivo experiments including chemogenetics, fluoxetine treatment, antagonist administration, and GRAB 5-HT sensor measurement. Blinding to single spine two-photon uncaging experiment was not possible because the researcher has to manually target and photo-stimulate a spine to determine the effect of stimulation on the structure and function of the target spines.

Reporting for specific materials, systems and methods

We require information from authors about some types of materials, experimental systems and methods used in many studies. Here, indicate whether each material, system or method listed is relevant to your study. If you are not sure if a list item applies to your research, read the appropriate section before selecting a response.

Materials & experimental systems

n/a	Involved in the study
☒	☐ Antibodies
☒	☐ Eukaryotic cell lines
☒	☐ Palaeontology and archaeology
☐	☒ Animals and other organisms
☒	☐ Clinical data
☒	☐ Dual use research of concern
☒	☐ Plants

Methods

n/a	Involved in the study
☒	☐ ChIP-seq
☒	☐ Flow cytometry
☒	☐ MRI-based neuroimaging

Animals and other research organisms

Policy information about studies involving animals; ARRIVE guidelines recommended for reporting animal research, and Sex and Gender in Research

Laboratory animals	Mice were acquired from Jackson Laboratories (C57BL/6NJ, wild type, and B6.129(Cg)-Slc6a4tm1(cre)Xz/J, SERT-Cre). Both male and female mice of both strains 1-4 weeks of age were used for all experiments.
Wild animals	No wild animals were used in the study.
Reporting on sex	Both male and female wild type and SERT-Cre mice were used.
Field-collected samples	No field collected samples were used in the study.
Ethics oversight	All the experiments involving animals were performed in accordance with the Institutional Animal Care and Use Committees of the University of Colorado on Anschutz Medical Campus (AAALAC Accredited #00235) and National Institutes of Health guidelines.

Note that full information on the approval of the study protocol must also be provided in the manuscript.

Plants

Seed stocks	N/A
Novel plant genotypes	N/A
Authentication	N/A
